# Maximum recovery after knee replacement – the MARKER study rationale and protocol

**DOI:** 10.1186/1471-2474-10-69

**Published:** 2009-06-17

**Authors:** Chung-Wei Christine Lin, Lyn March, Jack Crosbie, Ross Crawford, Stephen Graves, Justine Naylor, Alison Harmer, Stephen Jan, Kim Bennell, Ian Harris, David Parker, Helene Moffet, Marlene Fransen

**Affiliations:** 1 The George Institute for International Health, The University of Sydney, PO Box M201, Missenden Rd, Sydney NSW 2050, Australia; 2 Department of Rheumatology, Royal North Shore Hospital, St Leonards, NSW 2065, Australia; 3 Discipline of Physiotherapy, Faculty of Health Sciences, The University of Sydney, PO Box 170, Lidcombe NSW 1825, Australia; 4 Queensland University of Technology, 2 George St, Brisbane, QLD 4001, Australia; 5 Discipline of Public Health, University of Adelaide, Mail Drop 511, SA 5005, Australia; 6 Whitlam Orthopaedic Research Centre, Orthopaedic Department, Liverpool Hospital, Locked Bag 7103, Liverpool BC NSW 1871, Australia; 7 Centre for Health, Exercise and Sports Medicine, School of Physiotherapy, University of Melbourne, 200 Berkeley Street, Melbourne VIC 3010, Australia; 8 Director of Orthopaedics, Liverpool Hospital, Locked Bag 7103, Liverpool BC NSW 1871, Australia; 9 Sydney Orthopaedic Research Institute, Level 1, The Gallery, 445 Victoria Ave, Chatswood NSW 2067, Australia; 10 Department of Rehabilitation, Faculty of Medicine, Laval University Pavillon Ferdinand-Vandry, Room 4463, Québec, QC G1M 2S8, Canada; 11 Current address: Clinical and Rehabilitation Sciences Research Group, Faculty of Health Sciences, The University of Sydney, PO Box 170, Lidcombe NSW 1825, Australia

## Abstract

**Background:**

There is little scientific evidence to support the usual practice of providing outpatient rehabilitation to patients undergoing total knee replacement surgery (TKR) immediately after discharge from the orthopaedic ward. It is hypothesised that the lack of clinical benefit is due to the low exercise intensity tolerated at this time, with patients still recovering from the effects of major orthopaedic surgery. The aim of the proposed clinical trial is to investigate the clinical and cost effectiveness of a novel rehabilitation strategy, consisting of an initial home exercise programme followed, approximately six weeks later, by higher intensity outpatient exercise classes.

**Methods/Design:**

In this multicentre randomised controlled trial, 600 patients undergoing primary TKR will be recruited at the orthopaedic pre-admission clinic of 10 large public and private hospitals in Australia. There will be no change to the medical or rehabilitative care usually provided while the participant is admitted to the orthopaedic ward. After TKR, but prior to discharge from the orthopaedic ward, participants will be randomised to either the novel rehabilitation strategy or usual rehabilitative care as provided by the hospital or recommended by the orthopaedic surgeon. Outcomes assessments will be conducted at baseline (pre-admission clinic) and at 6 weeks, 6 months and 12 months following randomisation. The primary outcomes will be self-reported knee pain and physical function. Secondary outcomes include quality of life and objective measures of physical performance. Health economic data (health sector and community service utilisation, loss of productivity) will be recorded prospectively by participants in a patient diary. This patient cohort will also be followed-up annually for five years for knee pain, physical function and the need or actual incidence of further joint replacement surgery.

**Discussion:**

The results of this pragmatic clinical trial can be directly implemented into clinical practice. If beneficial, the novel rehabilitation strategy of utilising outpatient exercise classes during a later rehabilitation phase would provide a feasible and potentially cost-effective intervention to optimise the physical well-being of the large number of people undergoing TKR.

**Trial Registration:**

ACTRN12609000054213

## Background

The number of total knee replacements (TKR) performed each year is increasing rapidly in most developed countries [1-7]. In Australia, the total number of TKR operations performed has increased by 150% in the past decade [1]. Currently the mean age of patients undergoing TKR in Australia is 70 years, however the proportion of patients aged less than 65 years at the time of surgery has been increasing over the years reaching 32% in 2007 [1]. It is anticipated that the demand for TKR surgery will at least double again within the next decade and the average age at surgery will continue to decrease [3,8]. This trend is not only attributable to the aging of the population, increased prevalence of obesity and the expectation of a 'baby boomer' cohort less willing to accept the sedentary lifestyle, but also involves factors such as the evolution in implant design and surgical procedures resulting in better outcomes from TKR in relatively early stages of osteoarthritis (OA) [9]. It is readily apparent that the public health significance of TKR surgery is and will continue to be sizable.

Severe symptomatic knee OA is the main indication for almost all primary TKR surgery [1,6,9,10]. People affected by severe symptomatic knee OA experience significant functional limitation [11], and reduced lower limb muscle strength [12], aerobic fitness [13] and quality of life [14,15]. The need for rehabilitation following TKR is based on the contention that these deficits do not spontaneously resolve after surgery [16-19].

For the majority of patients, there are marked improvements after TKR in pain and physical function, with the maximum achievable benefit appearing around 6 months after surgery [20,21]. However, the magnitude of improvement is smaller than that demonstrated after total hip replacement surgery [21-23], and about 15% of patients still report moderate to severe pain a year after TKR despite no evidence of radiographic abnormalities [24]. Many years after TKR, patients are left with the lower limb muscle strength deficits evident prior to surgery [25-27] and with imbalances between knee extensor and flexor forces [25]. Poor ligamentous and neuromuscular control may result in destructive mechanical stresses and reduced control over anterior shear forces on the knee implant, potentially limiting implant longevity. It follows then, that muscle strengthening should be a major goal of rehabilitation. In support of this, achievement of greater knee extensor strength after TKR has been associated with improved physical function [26]. Younger people (<65 years of age) demonstrate less improvement after TKR [14] and have higher rates of revision surgery [10,28,29]. Effective rehabilitation may be particularly important in this younger group as they require prolonged implant longevity and are more likely to be in full-time employment or imposing greater physical demands on the knee implant.

In Australia, rehabilitation following TKR is mostly delivered in an outpatient setting by physiotherapists [30]. This rehabilitation commences immediately after discharge from the acute orthopaedic ward and is usually completed within 6 weeks, with resource-intensive one-on-one modes of delivery more common than class-based modes. However, evidence from randomised controlled trials suggests that such outpatient rehabilitation is no more effective than no treatment [31,32] or a home exercise programme [33,34]. One possible reason for the lack of demonstrable benefit is that the evaluated exercise programs did not specifically monitor or progress training intensity [31-34]. However training intensity is unlikely to be able to be progressed sufficiently to achieve meaningful changes in muscle strength in the first few weeks after TKR when patients are still experiencing the anaemia, pain and oedema inevitably associated with recovery from major orthopaedic surgery.

In support of our hypothesis, a recent trial provided initial evidence that a supervised exercise programme commencing two months after TKR resulted in improved function and reduced pain compared to a home exercise programme [35]. The clinical benefits were evident both at the end of the treatment period and six months after TKR. Apart from timing, another noticeable difference between this rehabilitation programme and those evaluated in other clinical trials was that the training intensity was closely monitored and each exercise had varying difficulty and complexity to allow progression. However, the rehabilitation programme was delivered on an individual basis so was resource-intensive.

The aim of the proposed clinical trial is to compare, the long-term effectiveness of a novel rehabilitation strategy versus current usual rehabilitative care received after discharge from the orthopaedic ward in patients who have undergone primary TKR. The novel rehabilitation strategy consists of an initial home exercise programme during the early recovery period followed by two months of outpatient exercise classes. A health economic evaluation, undertaken from the perspectives of the health sector and society, will be conducted alongside this clinical trial. We hypothesise that the novel rehabilitation strategy will lead to greater gains in physical well-being and be more cost-effective than usual care.

## Methods

The MARKER (Maximum Recovery after Knee Replacement) Study is a multicentre randomised controlled trial that will be conducted in 10 large public and private hospitals in Australia. Participants will be required to provide written informed consent prior to starting the study. Ethics approval has been obtained from the University of Sydney Human Research Ethics Committee and from the Ethics Review Committee (RPAH Zone) of the Sydney South West Area Health Service using the National Ethics Application Form . The MARKER Study has been registered at the Australian and New Zealand Clinical Trials Registry (ACTRN12609000054213).

Potential participants will be screened and recruited at the orthopaedic pre-admission clinic. Inclusion criteria are: (i) aged between 45 to 74 years, (ii) planned unilateral or bilateral primary TKR, and (iii) able to be discharged home from the orthopaedic ward. Exclusion criteria include: (i) previous unicompartmental replacement or tibial osteotomy on the same knee, (ii) previous lower limb joint replacement surgery within the last six months, (iii) further lower limb joint replacement surgery anticipated within the next 12 months, (iv) major co-morbidity precluding aerobic exercise at 50–60% maximum heart rate, (v) rheumatoid arthritis, (vi) major neurological conditions, or (vii) inability to return to one of the participating sites for outpatient exercise classes.

There will be no change to usual medical or rehabilitative care during the peri-operative inpatient period. After TKR, but before discharge from the orthopaedic ward, participants will be randomly allocated to either the novel rehabilitation strategy or usual rehabilitative care specific for the orthopaedic centre or surgeon (control group). The randomisation schedule will be generated a priori using a computer generated random number sequence by an independent researcher with no participant contact, in varying blocks of four or six, and stratified by recruitment site and by unilateral or bilateral TKR. Allocations will be sealed in opaque and consecutively numbered envelopes with a clear audit trail.

### Interventions

#### Novel rehabilitation strategy

Stage 1: Initial home exercise programme (usually Week 1 to 6 after randomisation)

Participants will be provided with a written home exercise programme on discharge from acute care (Additional file 1). The exercises will focus on maintaining and improving active knee flexion and extension range of motion. Participants will repeat the exercises 5 to 10 times per exercise, three times daily. Participants' progress, including adherence to the home exercise programme, walking ability and knee symptoms (e.g. pain, wound healing, range of motion, swelling), will be monitored weekly by a research physiotherapist, with clinic visits in the first one or two weeks and by telephone calls thereafter.

Stage 2: Outpatient exercise classes (usually Week 6 to 14 after randomisation)

Approximately five weeks after randomisation, the research physiotherapist will commence screening during the weekly telephone calls to assess eligibility to commence the classes: (i) surgical wound healed, (ii) full weight-bearing tolerated on the operated limb, (iii) ambulating independently outdoors for more than 50 m, and (iv) not requiring daily opioid-based analgesics for knee pain. Participants will join the outpatient exercise classes as they become eligible.

The classes will be circuit-based and supervised by a physiotherapist. The one hour classes will be conducted twice a week. Participants will be encouraged to attend the ongoing classes for eight weeks. Each class will consist of a short warm up and cool down component, progressive functional and strengthening exercises, and a 20-minute monitored aerobic exercise session on a stationary bicycle (Additional file 1). In order to standardise this programme across centres, minimal equipment will be used and supervising physiotherapists will receive prior training from research staff as well as a written manual of procedures. Participants will record the loads, repetitions and sets for to each functional and strengthening exercise they complete, and the duration and exertion (maximal heart rate or the Borg Rating of Perceived Exertion Scale [36]) for the aerobic exercise, in an exercise diary during the classes.

At the beginning of each exercise class, knee pain, range of motion and swelling will be evaluated to allow appropriate exercise progression. To allow this level of monitoring, class size will be restricted to a maximum of six participants. An education class will be provided once a month focusing on the rationale for increasing lower limb muscle strength and beneficial lifestyle behaviours appropriate after TKR. In addition, participants will be required to do at least one additional intensive exercise session per week at home, consisting of 30 minutes of walking outdoors or stationary bicycle.

#### Control group

Participants in the control group will receive the usual rehabilitation management as recommended by their orthopaedic surgeon or provided by the hospital, and can be delivered in the inpatient or outpatient setting. Findings from a previous Australian national survey demonstrate that rehabilitation will usually commence immediately after discharge from the orthopaedic ward, be completed within 6 to 8 weeks after TKR and will be provided on an individual basis by a physiotherapist in the outpatient setting [30]. Rehabilitation received by the control group will be documented in the patient diary.

### Outcomes assessments

Assessments will be conducted at baseline (pre-admission clinic), and 6 weeks, 6 months and 12 months after randomisation by assessors blinded to treatment allocation. Demographic information collected at baseline will include height, weight, socioeconomic data (highest level of education completed, health insurance status, employment status, occupation), history of lower limb arthritis and joint surgery and the 19-item Hospital for Special Surgery Knee Replacement Expectations Survey [37,38]. This survey has been tested for internal consistency (Cronbach's alpha = 0.77) and test-retest reliability [37].

#### Primary outcomes

The primary outcomes will be self-reported knee pain and physical function measured on the Western Ontario and McMaster Universities (WOMAC) Osteoarthritis Index, using a 5-point Likert scale (WOMAC LK3.1) [39]. The WOMAC consists of 24 items covering three subscales: pain (five items), stiffness (two items) and physical function (17 items). The pain and physical function subscale will be used, each subscale transformed to a score ranging from 0 (no pain or difficulty) to 100 (maximum pain or difficulty). The WOMAC is a widely-used questionnaire specifically designed to evaluate knee and hip OA [40,41]. Its clinimetric properties have also been validated in patients undergoing TKR [42-44].

Responses on the WOMAC will also be dichotomised according to the OMERACT-OARSI responder criteria for OA [45], i.e. treatment responders are defined as those participants achieving ≥20% improvement in both pain and physical function scores and an absolute change of ≥10, or ≥50% improvement in pain or physical function scores and an absolute change of ≥20 on the WOMAC.

#### Secondary outcomes

Secondary outcomes include:

• Quality of life (12-Item Short-Form Health Survey (SF-12) [46], Version 2). Data from the 12 items will be used to construct the physical and mental component summary scores. The SF-12 is a shorter questionnaire derived from the 36-Item Short-Form Health Survey (SF-36) and correlates well with the SF-36 (r > 0.94) [47].

• Priority for joint replacement surgery (Multi-attribute Arthritis Prioritisation Tool (MAPT) [48]). The MAPT is an 11-item questionnaire that assesses the need for joint replacement surgery. The questionnaire has high internal consistency (alpha = 0.85), test-retest reliability (ICC = 0.89) and correlation with the WOMAC (r = 0.78) [49].

• Participation in leisure time physical activity will be measured by the self-reported frequency and total duration over the past week of walking continuously, moderate physical activities and vigorous physical activities [50]. Participants will be categorised as participating in sufficient physical activity when more than 150 minutes of activity is accrued in at least five separate sessions per week [51].

• Fatigue ('how much of a problem has fatigue or tiredness been for you in the last week'). Responses will be marked on a 100-mm visual analogue scale, with anchor points being 'fatigue is no problem' and 'fatigue is a major problem' [52].

• Use of analgesics. The use of prescription and over-the-counter analgesics over the past week will be recorded, and the dosage adjusted for potency using defined daily dosage [53]

• Knee stability – the self-reported degree to which knee instability (partial giving way or full giving way) affects daily activities will be measured using the two stability items from the Activities of Daily Living Scale of the Knee Outcome Survey [54].

• Satisfaction with rehabilitation management ('how satisfied have you been with your rehabilitation programme since leaving the hospital where you had your surgery?') Responses will be required on a 6-point Likert scale, ranging from 'completely dissatisfied' to 'completely satisfied'.

• Walking speed (50-foot (15.24 m) timed walk [55]). Participants will be asked to walk as fast as possible within the limits of safety, with or without a walking aid.

• Knee range of motion. The centre of an extendable goniometer will be positioned over the lateral knee joint space with the arms aligned with the lateral midline of the femur using the greater trochanter as reference, and the lateral midline of the fibula using the head of fibula and lateral malleolus as reference [56]. Knee flexion will be measured as an active movement in sitting. Knee extension will be measured as an active-assisted movement in long sitting or supine with the heel supported the plinth during the movement.

• Lower limb power (stair climb test). Power on stair climb will be calculated using a formula that incorporates body mass, the time taken to ascend 10 stairs and total stair height [57]. This is a reliable and responsive measure in people with knee OA undergoing TKR [17].

• Maximal isometric knee flexion and extension strength. With the patient seated and the knee flexed at 90 degrees, a 'make test' will be performed against a handheld dynamometer, and the best of three attempts recorded. Strength will only be measured at the 6- and 12-month follow-up assessments because participants will be required to comfortably achieve 90 degrees of knee flexion.

The occurrence of adverse events will be sought from participants at each follow-up assessment. Adverse events will be defined as any musculoskeletal or cardiovascular event resulting in medical intervention or reduced function for three or more days. An independent Data Safety and Monitoring Committee (DSMC), comprising of experts in clinical trials, biostatistics and rehabilitation will be established to review data on adverse events after 300 study participants (half the planned study sample) have completed the six-month follow-up assessment. The DSMC will be charged with informing the study investigators if evidence emerges beyond reasonable doubt that treatment allocation is associated with an increased risk of serious adverse events that would be expected to change the practice of clinicians responsible for the rehabilitation of patients after primary TKR surgery.

### Health economic outcomes

Participants will prospectively record in a patient diary the following:

• presenteeism (self-reported capacity to work or perform daily activities) [58-60]

• instances of absenteeism

• use of healthcare and community services

• out-of-pocket costs of community services

These data will be collected over three six-week time periods from: (i) discharge from acute care, (ii) the 6-week follow-up assessment, and (iii) the 6-month (26-week) follow-up assessment.

Presenteeism will be rated from 0% (unable to do usual work/activities) to 100% (fully functioning). Absenteeism is defined as days off work (including home, volunteer or carer duties) due to TKR. The use of healthcare (e.g. visits to medical practitioners, physiotherapists, hospitalisation) and community (e.g. meals on wheels) services will be recorded in terms of the type of service, date of use and number of hours/days. Costs of community services will be the out of pocket costs incurred by participants. The use of medication will be included as a health economic outcome and will be collected at the time of each follow-up assessment.

### Five-year follow-up

All participants will be asked to consent to a telephone call to complete the WOMAC, SF-12 and MAPT annually until five years after TKR. In addition, data linkage with the Australian National Joint Replacement Registry will be used to verify incident primary or revision hip or knee replacement surgeries occurring in our cohort during this five year period.

### Sample size

Based on preliminary data [35], a sample size of 600 participants will be required. This sample size will provide 80% power of detecting a 15% difference in the proportion of treatment responders (OMERACT-OARSI responder criteria [45]) between the groups, and allows for up to 10% cross-over of treatment allocation and a 15% loss to follow-up [61]. In addition, this sample size will provide more than 90% power to detect clinically significant differences in each of the continuous secondary outcome measures.

### Statistical analysis

Statistical analysis will be by intention to treat and coded to ensure blinding to treatment allocation. For continuous data, two-way repeated measures ANOVA incorporating treatment groups at the three follow-up time points (6 weeks, 6 months and 12 months) will be carried out. The significance of any differences in dichotomous data (OMERACT-OARSI treatment responders, proportion of patients reporting sufficient leisure time physical activity, number of adverse events) will be tested using generalised estimating equations or linear mixed model. If there are chance imbalances in baseline patient characteristics hypothesised to influence the main outcomes, then statistical techniques that allow adjustment for confounding variables will be used as secondary analyses. If there are more than 5% missing data, sensitivity analysis allowing for different assumptions such as the best or worst possible scenario will also be reported for the main outcomes of the study.

### Health economic analysis

The economic evaluation will consist of a trial-based cost-utility analysis to assess the incremental cost per quality-of life-year (QALY) of the novel rehabilitation strategy compared to usual care. The primary analysis will be conducted from the perspective of the health sector. A secondary analysis will entail a societal perspective in which the additional costs (and cost savings) associated with use of community services, absenteeism and presenteeism will be factored in.

In the primary analysis, costs of healthcare services will be valued at standard rates published by the Australian Government: the Medical Benefits Scheme standard fees for medical services and procedures, the Pharmaceutical Benefits Scheme cost for medications and the Australian Refined Diagnosis Related Groups (AR-DRG) cost weights for hospital services. These will be factored with levels of utilisation to estimate the costs of healthcare use in participants.

In the secondary analysis, costs of community services will be based on the self-reported costs of participants. The costs of presenteeism will be estimated by the number of days worked weighted by the rate of presenteeism multiplied by the average wage rate for the relevant employment category. The costs of absenteeism will be estimated by the number of days absent from work multiplied by the average wage rate for the relevant employment category. For those engaged in unpaid work, the national average wage rate will be used. A separate set of cost-utility ratios will be presented in this secondary analysis incorporating the wider costs (and cost savings) to the community.

Health state utilities required to estimate QALY will be based on measures obtained from the SF-12 and transformed into health state utilities via the SF-6D algorithm [62]. Although no survival difference is expected between the two groups at 12 months, average quality of life over 12 months for each participant will be estimated and weighted by survival up to 12 months to determine, for each patient, a measure of QALY after TKR. Given likely variations in the 'usual care' across sites, separate incremental cost-utility ratios will be estimated for broad categories of usual care (inpatient or outpatient). Sensitivity analysis will test uncertainty in key parameters such as the selection of cost weights and statistical variation in quality of life scores.

## Discussion

The primary aim of the proposed pragmatic randomised controlled trial is to compare the effectiveness and cost-effectiveness of a novel rehabilitation strategy, consisting of an initial home exercise programme followed by higher intensity outpatient exercise classes, to usual rehabilitative care in people undergoing primary TKR for OA. The trial incorporates methodological features that have been shown to reduce bias [63-66]. Participants will be assigned to the novel rehabilitation strategy or usual care by random, concealed allocation with an audit trail. Outcomes assessments will be blinded. The nature of the treatment precludes blinding of the therapist and the participant, but statistical analysis will be blinded to treatment allocation and will be conducted in accordance with 'intention to treat' methods.

Current evidence does not support the usual practice of providing outpatient rehabilitation in the period immediately following discharge from the acute orthopaedic ward after TKR in terms of improving self-reported of physical outcomes. In light of the increasing incidence of TKR, the need to develop clinically effective and cost-effective rehabilitation programs for the growing number of people undergoing TKR surgery is paramount.

The MARKER Study is a pragmatic randomised controlled trial designed with sufficient power to detect meaningful clinical and economic benefits if they exist. The evaluated rehabilitation programme is class-based, utilises only simple and widely available equipment and can therefore be easily implemented in clinical practice. If effective, the novel rehabilitation strategy could optimise outcomes in people after TKR and allow substantial cost savings to both the health sector and more widely across the community when compared to the usual, resource-intensive one to one outpatient intervention. Furthermore, our own observations indicate access to early outpatient rehabilitation is affected by a patient's impaired physical state and driving restrictions. A programme that allows home exercise in the early recovery period followed weeks later by outpatient rehabilitation circumvents this access problem, thus potentially facilitating improved patient adherence to a prescribed supervised programme.

Currently 10 large orthopaedic centres and associated physiotherapy outpatient departments have pledged collaboration. It is anticipated that the MARKER Study will commence patient recruitment in early 2009. The one-year follow-up of all study participants should be completed by the end of 2011.

## Competing interests

The authors declare that they have no competing interests.

## Authors' contributions

CL contributed to the design of the study and drafted the manuscript. LM, JC, RC, SG, JN, AH, SJ, KB, IH, DP, HM contributed to the design of the study. SJ designed the health economic evaluation. IH participated in recruitment of study centres. MF conceived and designed the study, contributed to the manuscript and leads the MARKER Study Collaboration. All authors read and approved the final manuscript.

## Pre-publication history

The pre-publication history for this paper can be accessed here:



## Supplementary Material

Additional file 1 **Appendix 1**. The MARKER Study: Novel Rehabilitation Strategy. Click here for file
