# Supplementary material for: Maximum recovery after knee replacement – the MARKER study rationale and protocol
Source: BMC Musculoskelet Disord. 2009 Jun 17;10:69. doi: 10.1186/1471-2474-10-69 (PMC2709653; doi:10.1186/1471-2474-10-69)
Supplement: Additional file 1 — Appendix 1. The MARKER Study: Novel Rehabilitation Strategy. [file 1471-2474-10-69-S1.doc]

**Appendix I**

**The MARKER Study**

**Novel Rehabilitation Strategy**

**Stage 1: initial home exercise programme**

- Commencing immediately after discharge from the acute orthopaedic ward
- Also includes weekly monitoring and individualised advice from the physiotherapist on swelling, pain, and walking

**Weeks 1 to 2 after randomisation**

- Active assisted knee extension and flexion range of motion in sitting
- Inner range quadriceps strengthening
- Straight leg raise

**Weeks 2 to 6 after randomisation**

- Active knee flexion and extension range of motion in sitting
- Passive knee flexion range of motion on step
- Passive knee extension stretch
- Inner range quadriceps strengthening
- Sitting to standing from a high surface/chair, progressing to chair of usual height

**Stage 2: outpatient exercise classes**

- Commencing when participants satisfy the following criteria: (i) surgical wound healed, (ii) full weight-bearing tolerated on the operated limb, (iii) ambulating independently outdoors for more than 50 m, and (iv) not requiring daily opioid-based analgesics for knee pain.
- In the first week of the class programme, the aim will be to familiarise participants with the programme and allow one hour of exercise at a comfortable intensity.

**Warm up** (5 to 10 minutes)

- Knee active range of motion in sitting
- Walking in gym. Substitute with stepping on the mini-trampoline in later weeks.
- Hamstring and calf stretches

**Functional and strengthening exercises** (30 minutes)

- Exercises performed at three sets of 8 to 12 repetitions from Week 3 of the class programme [1]
- The functional and strengthening exercises form a circuit of six stations. Participants will have approximately five minutes at each station
- Unilateral exercises will be repeated on both legs
- Progression can be made by increasing resistance (provided by weight belts or body weight), step height (for exercises on stairs) or width of step (for lunges), or progressing to the next exercise within the station.

Stations

A. Quadriceps and step-out

1. Inner range quadriceps strengthening in supine
2. Quadriceps strengthening in sitting
3. Stepping out to different directions in standing

B. Stairs

1. Step up
2. Side step
3. Step down
4. (Alternative to 2 and 3) Step-through gait pattern on stair ascent and descent

C. Squats

1. Squats with both legs
2. Sitting to standing
3. Squats against the wall

D. Hip exercises

1. Hip abduction and extension strengthening in standing
2. Bridging

E. Lunges

1. Stationary lunges
2. Walk lunges

F. Power – the focus for this station is on low intensity and fast repetition

– All of these exercises are performed each time: step up, sitting to standing from a high surface, calf raises

**Aerobic exercise on the stationary bicycle** (20 minutes)

A heart rate monitor will be used and participants will exercise within a training heart rate zone based on the Karvonen formula [2]:

Training heart rate zone (b·min^-1^) = (HR_max_ – HR_rest_) x training intensity + HR_rest_

where HR_max_ = estimated maximum heart rate, 220 – age

HR_rest_ = resting heart rate

HR_max_ – HR_rest_ = heart rate reserve (HRR)

Training intensity will start at 50 to 60% at Week 3 and gradually increase to 60 to 70%.

For participants taking beta-blockers, the Borg Rating of Perceived Exertion (RPE) scale [3] will be used to monitor aerobic exercise intensity. The Borg RPE ranges from 6 (no exertion at all) to 20 (maximal exertion). Participants will aim to achieve ratings of 12 to 13 on the Borg RPE, which corresponds to moderate intensity of exercise and 55-69% of maximal heart rate [1].

**Cool down** (5 to 10 minutes)

- Walking in gym. Substitute with stepping on the mini-trampoline in later weeks.
- Hamstring and calf stretches

**References**

1. Pollock ML, Gaesser GA, Butcher JD, Després J-P, Dishman RK, Franklin BA, Garber CE: **American College of Sports Medicine Position Stand. The recommended quantity and quality of exercise for developing and maintaining cardiorespiratory and muscular fitness, and flexibility in healthy adults**. *Med Sci Sports Exerc* 1998, **30**:975-997.

2. Karvonen MJ, Kentala E, Mustala O: **The effects of training on heart rate; a longitudinal study**. *Ann Med Exp Biol Fenn* 1957, **35**:307-315.

3. Borg G: **Perceived exertion as an indicator of somatic stress**. *Scand J Rehabil Med* 1970, **2**:92-98.
